# *TERT* promoter hotspot mutations in breast cancer

**DOI:** 10.1007/s12282-017-0825-5

**Published:** 2017-12-08

**Authors:** Tatsunori Shimoi, Masayuki Yoshida, Yuka Kitamura, Tomomi Yoshino, Asuka Kawachi, Akihiko Shimomura, Emi Noguchi, Mayu Yunokawa, Kan Yonemori, Chikako Shimizu, Takayuki Kinoshita, Koichi Ichimura, Takahiro Fukuda, Yasuhiro Fujiwara, Kenji Tamura

**Affiliations:** 1 Department of Breast and Medical Oncology, National Cancer Center Hospital, 5-1-1 Tsukiji, Chuo-ku, Tokyo, 104-0045 Japan; 2 Graduate School of Medicine, Course of Advanced Clinical Research of Cancer, Juntendo University, Tokyo, Japan; 3 Department of Pathology and Clinical Laboratories, National Cancer Center Hospital, Tokyo, Japan; 4 Department of Breast Surgery, National Cancer Center Hospital, Tokyo, Japan; 5 Division of Brain Tumor Translational Research, National Cancer Center, Tokyo, Japan; 6 Department of Hematopoietic Stem Cell Transplantation, National Cancer Center Hospital, Tokyo, Japan

**Keywords:** Breast cancer, Telomerase reverse transcriptase, *TERT* promoter mutation

## Abstract

**Background:**

Telomerase reverse transcriptase (*TERT*) promoter mutations have been discovered in solid and hematological malignancies, where they reflect *TERT* activation and cell-cycle progression. In melanoma, glioma, and thyroid cancers, *TERT* promoter mutations are associated with a poor prognosis. However, no studies have evaluated the prevalence and prognostic significance of *TERT* promoter mutations in breast cancer.

**Methods:**

We analyzed *TERT* promoter hotspot mutations (C228T and C250T) using direct sequencing of DNA from 319 tumor tissues. We also collected clinical data from cases that were positive for *TERT* promoter mutations.

**Results:**

We detected *TERT* promoter mutations in three (0.9%) of the 319 cases. Two patients had hormone receptor-positive and human epidermal growth factor receptor 2-negative cancer, while the third patient had triple-negative cancer. All three patients had initially been diagnosed with operable breast cancer and undergone surgical treatment. The relapse-free survivals of these patients were 83, 226, and 270 months, respectively. The mutations were C250T in the triple-negative cancer case and C228T in the remaining two cases.

**Conclusion:**

Given the rarity of *TERT* promoter mutations, further studies are needed to confirm their prognostic significance in breast cancer cases.

## Introduction

Breast cancer is a common cancer and the leading global cause of cancer-related mortality among women [1]. In addition, breast cancer is a heterogeneous condition that is categorized into four subtypes according to pathological review, hormone receptor status, and human epidermal growth factor receptor 2 (HER2) status. Furthermore, using whole-genome sequencing, numerous somatic and driver mutations have been identified in breast cancer [2]. This information has allowed physicians to stratify patients according to their tumor’s molecular characteristics and select appropriate therapies. Recent clinical trials have indicated that mutations in the genes for phosphatidylinositol-4,5-bisphosphate 3-kinase catalytic subunit alpha (*PIK3CA*) and RAC-alpha serine/threonine-protein kinase (*AKT1*) may predict the response to PI3 K and AKT inhibitors, respectively [3–5].

Telomerase is a DNA polymerase that maintains the length of telomeres at the end of chromosomes. Its activity is relatively high in stem cells and is downregulated in normal somatic cells. However, genetic mutations can also affect the non-coding regulatory region of the telomerase reverse transcriptase (*TERT*) gene’s promoter, and telomerase can be activated by mutations in the *TERT* promoter. Many malignant tumor cells have *TERT* expression or telomerase activity, with > 90% of breast cancer cases having telomerase activity [6].

In 2013, somatic hotspot mutations in the promoter region of *TERT* were reported among patients with melanoma [7, 8]. In addition to these two common hotspots identified on chromosome 5: 1,295,228 C > T (C228T) and 1,295,250 C > T (C250T), the tandem mutations of 1,295,228/1,295,229 CC > TT (C228T/C229T) and 1,295,242/1,295,243 CC > TT (C242T/C243T) have also been reported in melanoma with *TERT* promoter mutation. In a previous report, mutation frequencies for C228T, C250T, C228T/C229T and C242T/C243T in melanoma cell lines were 46/168 (27%), 64/168 (38%), 7/168 (4.2%) and 8/168 (4.8%), respectively. On the contrary, no mutation sites other than C228T and C250T have been reported in glioma. In one large retrospective cohort, the mutation frequencies of C228T and C250T in primary glioblastoma with *TERT* promoter mutation were 123/256 (48%) and 56/256 (22%), respectively [13]. Since the first report of melanoma, > 30 types of tumors have been found to contain *TERT* promoter mutations, hepatocellular carcinoma (41%), thyroid cancer (11–43%), ovarian clear cell carcinoma (16%), bladder cancer (63%), and phyllodes tumors of the breast (65%) [9–11]. In these types of cancer other than melanoma, C228T and C250T were also hotspots of *TERT* promoter mutations, in which C228T is more dominant than C250T. These hotspot mutations are associated with a poor prognosis [7, 12, 14, 15]. However, to the best of our knowledge, no reports have evaluated the prevalence and prognostic significance of *TERT* promoter mutations in human breast cancer. Therefore, the present study aimed to provide information regarding *TERT* promoter mutations in samples from human breast cancer.

## Materials and methods

We retrospectively evaluated 319 breast cancer specimens (125 frozen specimens and 194 formalin-fixed paraffin-embedded [FFPE] specimens) that were provided by the National Cancer Center Biobank of Japan and had been obtained between January 1983 and November 2015. Three patients had metachronous breast cancer and we included all of their samples in this study. We extracted DNA from the frozen specimens using the QIAamp DNA FFPE tissue kit (QIAGEN, Tokyo) and from the FFPE specimens using the QIAamp DNA Mini Kit (QIAGEN, Tokyo), according to the manufacturer’s recommendations. The *TERT* promoter mutations (C228T and C250T) were identified using direct sequencing according to the previously reported methods [12, 13], and the direct sequencing was performed by FASMAC (Tokyo, Japan). The forward primer’s sequence was 5′-gtaaaacgacggccagcaggaaacagctatgacccagctccgcctcctccg-3′ and the reverse primer’s sequence was 5′-gctgcctgaaactcgcgcc-3′. Mutations in the *PIK3CA* gene (E542K, E545K, or H1047R) were detected using quenching probe system by the i-densy IS-5320 system (ARKRAY Inc., Kyoto, Japan) [16].

Estrogen receptor (ER) and progesterone receptor (PgR) were classified positive if ≥ 10% of tumor cells demonstrated positive nuclear staining for ER or PgR, respectively [17]. HER2 positivity was classified according to the recommendation of the American Society of Clinical Oncology/College of American Pathologists guidelines [18]. Hormone receptor positive was defined as positive for ER or PgR. Histological and nuclear grades were reported according to previously reported criteria [19, 20].

Relapse-free survival was defined as the time between the day of diagnosis and disease progression or last follow-up. Overall survival was defined as the time between the day of diagnosis and the day of death or last follow-up.

This study’s retrospective protocol was approved by the National Cancer Center Institutional Review Board (No. 2014-092). Written informed consent was not obtained from the patients. The results of this study have been published on our hospital’s web page.

## Results

**Fig. 1:**
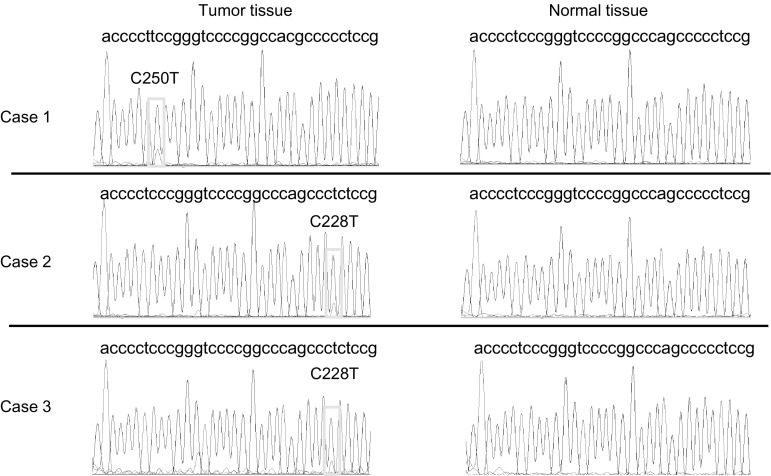
*TERT* promoter mutations in the three breast cancer cases. Sanger sequencing of the tumor tissue DNA revealed C250T (case 1) and C228T (cases 2 and 3) somatic mutations in the *TERT* promoter of the three breast cancer patients. All patients demonstrated *TERT* promoter mutations only in their tumor tissue DNA and not in the normal tissue DNA. Positive result for a *TERT* hotspot mutation is indicated by the corresponding yellow box

Sequencing was successful for all 319 specimens, and we detected *TERT* promoter mutations in three samples (0.9%). The *TERT* promoter mutations were only detected in the tumor tissue DNA and were not detected in the three patients’ normal tissue DNA. The three patients’ characteristics are shown in Table 1. All patients were women who had experienced a relatively late relapse, after undergoing surgery because of an early diagnosis of breast cancer. Two patients demonstrated relapse-free survivals of > 200 months. The histological results were invasive ductal carcinoma in two cases and invasive lobular carcinoma in one case. Two patients had hormone receptor-positive and HER2-negative breast cancer, while one patient had triple-negative breast cancer (TNBC). C250T mutations were noted in the TNBC case and C228T in the two hormone receptor-positive cases (Fig. 1). Two of these patients had mutations in the *PIK3CA* kinase domain (H1047R) (Table 1).

**Table 1:** The characteristics of the three breast cancer patients with *TERT* promoter mutations

	Case 1	Case 2	Case 3
Age, years	59	41	46
Sex	Female	Female	Female
Initial stage	1A	ND	2A
Histology	IDC	IDC	ILC
Histological grade	2	2	1
Nuclear grade	1	2	1
Estrogen receptor (AS)	Negative (0)	Positive (8)	Positive (8)
Progesterone receptor (AS)	Negative (0)	Positive (8)	Positive (8)
HER2 status (IHC score)	Negative (0)	Negative (0)	Negative (1+)
*PIK3CA* hotspot mutation	H1047R	Negative	H1047R
*TERT* hotspot mutation	C250T	C228T	C228T
Relapse-free survival, months	83	226	270
Overall survival, months	100	446	300
Status	Alive	Alive	Alive

*ND* no data, *IDC* invasive ductal carcinoma, *ILC* invasive lobular carcinoma, *AS* Allred score, *HER2* human epidermal growth factor receptor 2, *IHC* immunohistochemistry

## Discussion

This is the first detailed report regarding *TERT* promoter mutations in human breast cancer, and we identified *TERT* promoter mutations in three (0.9%) of 319 samples. However, a previous report identified two cases with *TERT* promoter mutations among eight human breast cancer cell lines (25%) [12]. Nevertheless, another report failed to detect *TERT* promoter hotspot mutations in 88 breast cancer samples [10]. In the present study, we found that two of the three samples with *TERT* promoter mutations also had *PIK3CA* kinase domain mutations. In ovarian cancer, *TERT* promoter mutation and *PIK3CA* mutation are considered mutually exclusive [21], whereas this coexistence is reported in other carcinomas [22, 23]. Given that *PIK3CA* mutation is a relatively common mutation in breast cancer, we investigated the coexistence of these mutations. Interestingly, *PIK3CA* mutations only exist in approximately 30% of patients with hormone receptor-positive breast cancer and in approximately 10–20% of TNBC cases. Moreover, *TERT* promoter mutations coexist with *PIK3CA* mutations in 12% of anaplastic thyroid cancers [22], but only in 7.5% of gliomas [23]. Thus, it is possible that a relationship exists between *TERT* promoter and *PIK3CA* kinase domain mutations.

*TERT* promoter mutations occur during the early stage of glioma or thyroid cancers and are associated with a poor prognosis among patients with melanoma, glioma, and thyroid cancer [22, 24–26]. Moreover, a recent study showed that the prognostic effect of *TERT* promoter mutation was different for each melanoma subtype [27]. Our study has few samples; hence, it is difficult to be definite about the prognosis. However, we could not conclude that the *TERT* promoter mutation was a poor prognostic factor based on our results.

It would have been indeed useful to analyze telomerase activity and/or *TERT* expression in these tumors. However, fresh-frozen tissues would be required to assess either of them. Unfortunately, materials available for this study were only FFPE specimen from the biopsy, and no frozen tissues were preserved. We could not extract enough RNA from the FFPE tissue samples, and no antibody against *TERT* for immunohistochemistry is currently available. Therefore, it was impossible to examine telomerase activity or *TERT* expression. In most previous reports, *TERT* expression was significantly elevated in cases with unified hotspot mutation compared with the wild-type cases [10, 12–14, 21, 25, 28, 29]. We believe our mutated tumors had elevated *TERT* expression.

Further studies are needed to clarify our findings. We consider two types of prospective cohort studies to investigate the prognostic importance and predictive factor in breast cancer. In one large-scale cohort study, which include prognostic factors of breast cancer such as stage, age, subtype, grade, or other biomarkers with *TERT* promoter mutation, we will be able to investigate whether the *TERT* promoter mutation is a prognostic factor. On the contrary, a study on ovarian cancer reported that a high *TERT* expression is a predictive factor for the effect of eribulin mesylate [30]. Therefore, another large-scale cohort study may also consider whether the *TERT* promoter mutation is a predictive factor of the effect of an anticancer agent, especially eribulin mesylate.

In conclusion, we have demonstrated the presence of *TERT* promoter mutation in breast cancer.
